# Infection prevention and control practices during the COVID-19 pandemic: impact on the prevalence of hospital-acquired infections in Tunisia

**DOI:** 10.1017/ash.2025.10125

**Published:** 2025-09-17

**Authors:** Salma Balhi, Héla Ghali, Bouthaina Hamza, Nihel Haddad, Houyem Said, Sana Bhiri, Asma Ben Cheikh

**Affiliations:** 1 University of Sousse, Faculty of Medicine of Sousse, Sousse, Tunisia; 2 Department of Preventive Medicine and Community Medicine, Sahloul University Hospital, Sousse, Tunisia

## Abstract

**Background:**

During the coronavirus disease 2019 (COVID-19) pandemic, strict infection prevention and control (IPC) measures were implemented in healthcare settings.

**Aim:**

To evaluate the effect of enhanced IPC practices implemented during the COVID-19 pandemic on the prevalence of hospital-acquired infections (HAIs) in a Tunisian university hospital.

**Methods:**

A multimodal IPC strategy was implemented from March to September 2020 at Sahloul University Hospital, Sousse, Tunisia. This strategy included promoting hand hygiene (HH), enhancing adherence to standard precautions and universal masking. We compared the prevalence of HAIs and compliance with IPC practices before the pandemic (pre-intervention phase) and during the pandemic (post-intervention phase).

**Results:**

A total of 306 patients were surveyed in 2019 and 228 in 2021. The prevalence of HAIs increased slightly from 9.4% to 10.08% (*p* = 0.814). HH compliance improved significantly from 38.3% to 51.6% and sharp waste sorting compliance rose from 60.3% to 77.6%. The use of personal protective equipment also increased, with FFP2 respirator consumption increasing nearly 247-fold.

**Conclusions:**

This study found no significant change in HAI prevalence despite the implementation of enhanced IPC strategies. However, the small sample size was a major limitation that may have reduced the statistical power to detect meaningful differences. Larger-scale studies are needed to confirm these findings and better assess the impact of IPC strategies in this context.

**Trial registration:**

Not applicable.

## Background

Hospital-acquired infections (HAIs) are defined as infections contracted by patients during their care that were neither present nor incubating at the time of admission.^1^HAIs are an increasing public health concern.^1^ They represent one of the most common adverse events in healthcare settings ^1^and contribute to increased morbidity, mortality, and healthcare costs.^1^ Between 2022 and 2023, a point prevalence survey (PPS) conducted in Europe revealed that 7.1% of patients had at least one HAI.^2^ In Africa, a recent meta-analysis found that the pooled prevalence of HAI ranged from 7.24% to 28%, which was more than twice that observed in developed countries.^3^

According to the World Health Organization (WHO), HAIs are a consequence of inadequate healthcare quality, often resulting from the absence of an infection prevention and control (IPC) program, insufficient hand hygiene (HH) training, and inadequate infrastructure.^1^ However, up to 50% of HAIs could be prevented through adherence to IPC recommendations.^1^ Strengthening IPC strategies at both national and healthcare facility levels is crucial to preventing HAIs, including outbreaks of highly transmissible diseases.^1-4^

The COVID-19 pandemic posed significant challenges to healthcare systems.^5-7^ This period underscored the critical role of IPC measures in preventing the transmission of SARS-CoV-2 within healthcare settings.^8^The widespread transmission of SARS-CoV-2 led to high hospitalization rates and affected the availability of personal protective equipment (PPE) and work processes.^5–7^ Healthcare facilities had to rapidly adapt to manage an unexpected and significant influx of patients.^7^

However, the impact of COVID-19 preventive measures on HAI rates remains limited and a subject to debate.^9,10^ On the one hand, some studies suggest that the pandemic contributed to an increase in HAI rates, particularly in intensive care units, due to PPE supply shortage and facility capacity limitations, which compromised infection control standards.^11^ Additionally, the widespread and often high selective use of antibiotics among patients facilitated the emergence of antimicrobial resistance.^11,12^ On the other hand, others have argued that enhanced hygiene practices, increased use of PPE, adherence to standard precautions and improved environmental cleaning, originally implemented to contain the transmission of SARS-CoV-2, had a beneficial impact on HAI prevention.^13–15^

In Tunisia, HAIs are also a major public health issue.^16^ The national Noso-Tun2012 survey, conducted in 144 public and private healthcare facilities, estimated the prevalence of HAIs at 7.2%.^16^ Like many other countries, to limit the spread of COVID-19, Tunisia implemented enhanced preventive measures, both in the general population and in healthcare settings.^17^ These measures included social distancing, the use of hand sanitizers with at least 60% alcohol content, and mask-wearing.^17^ A Tunisian study involving 723 frontline healthcare workers even found that PPE was overused during the pandemic.^18^ However, the impact of these protective measures on HAI prevalence in Tunisia remains unclear and requires further investigation.

Therefore, the main objective of this study was to evaluate the effect of enhanced IPC practices implemented during the COVID-19 pandemic on the prevalence of HAIs in a Tunisian university hospital. Second, we assessed compliance with IPC measures during the same period.

## Methods

### Study design

A pre-experimental study was conducted at Sahloul University Hospital in Sousse, Tunisia, over a 3-year period (2019–2021), with 2019 representing the pre-intervention phase and 2021 corresponding to the post-intervention phase.

Sahloul University Hospital is a tertiary teaching facility, with a surgical focus, located in central Tunisia. It has 654 active beds and recorded 25,969 inpatient admissions in 2024. The study was approved by the local Ethics Committee.

### Participants

All patients who had been hospitalized for 48 hours or more across all hospital wards (units or departments) and had not been discharged from the ward at the time of the survey were included. The following were excluded:

- SARS-CoV-2 suspected or confirmed positive cases, which were isolated and admitted to wards or specialized critical care units.
- Patients from the emergency department, hemodialysis and endoscopy units, due to short stays.

### Intervention: infection prevention and control strategy implemented during COVID-19 pandemic in Sahloul hospital

From the declaration of the first case of SARS-CoV-2 infection in Tunisia, on March 4, 2020, a COVID-19 committee was established at Sahloul University Hospital. This committee progressively implemented prevention and control strategies to mitigate the risk of coronavirus transmission within the hospital. These preventive measures included the following:

- Separation of patient pathways into “COVID-19” and “Non-COVID-19” zones.
- Establishment of two main triage stations outside the hospital building (for temperature checks and screening of the COVID-19 symptoms).
- Restriction of visitor numbers.
- Suspension of non-emergency medical services such as elective surgeries.
- Introduction of a universal masking policy for all healthcare workers in the hospital.
- Hospitalization of patients with respiratory symptoms in isolated wards.
- Reinforcement of regular HH using alcohol-based hand rub.

Moreover, The Preventive and Community Medicine Department implemented various bundles for HAI prevention including:

#### Active epidemiological surveillance

Continuous monitoring was conducted to promptly detect and respond to cases. A database tracking COVID-19 cases has been regularly updated since the hospitalization of the first confirmed COVID-19 patient in September 2020. All new cases of COVID-19 hospitalized in various units have been systematically registered. Additionally, daily monitoring of patients’ conditions was ensured by hygiene technicians from the department.

#### Training of at-risk workers

Theoretical and practical training sessions were conducted in person at the Sahloul Hospital Training Center. The program included educational presentations, video screenings, and interactive discussions with healthcare professionals. These sessions aimed to ensure mastery of and compliance with hospital protective measures against the risk of COVID-19 contamination. Training topics covered good HH practices (10 training sessions on HH over the first wave of the COVID-19 period from March 17 to March 26, 2020, were conducted), contact precautions for infected or colonized patients, prevention of COVID-19 transmission, proper donning and doffing of PPE, environmental cleaning for isolation units treating COVID-19 patients as well as healthcare waste management.

*Drafting guides, procedures, and protocols*: Comprehensive documents and videos were developed to standardize and guide infection control practices, such us the organization of septic isolation or the proper use of surgical and FFP2 masks.

*Assessment of professional practices*: regular audits were conducted to evaluate compliance with IPC measures, including HH audit of healthcare professionals, audits of bio-cleaning practices, and audits of sanitary healthcare waste management practices.

### Outcome measures

The primary outcome was to assess potential differences in HAI rates between patients hospitalized during the pre-intervention phase (2019) and those during the COVID-19 pandemic (2021), following the implementation of the IPC strategy.

The secondary outcome was to evaluate compliance with the IPC program, specifically HH compliance, audits of healthcare waste management practices, and the consumption of PPE, including surgical masks, FFP2 respirators, disposable gloves, and alcohol-based hand rub.

### Data collection and management

#### Point prevalence survey

The evaluation was based exclusively on annual PPS surveys, as no active surveillance system for HAIs was available duringthe study years (2019 and 2021). The PPS is conducted annually in March and early April to minimize the influence of seasonal variations in HAIs. Data were collected by the Preventive and Commnity Medicine Department investigator team. Each PPS was conducted on a single day, with one visit per hospital ward. In some cases, return visits were made to include patients hospitalized for at least 48 hours who were temporarily absent due to procedures or surgery. All data were collected and finalized within 2 weeks of the survey date. Data were gathered from medical records, laboratory reports, temperature charts, radiographs, and consultation with the referring health professionals, using a standardized form inspired by the “NosoTun 2012” survey tool.^19^ This form included five sections: admission data, demographic data, clinical data, antimicrobials (AM) use, and HAI data. HAI diagnosis were based according to the latest Centers for Disease Control and Prevention definition.^20^ All types of HAIs present during the study period were recorded and categorized.

### Data on compliance with IPC enhanced measures

#### Hand hygiene compliance

Annual HH audits were conducted in 2019 and 2021, respectively, by the investigator team from the Department of Preventive and Community Medicine. All hospital units were included except operating rooms, laboratories, and outpatient clinics. The audit targeted healthcare professionals—including physicians, nurses, and technicians—working in the selected departments who had direct patient contact and were present during the observation rounds. Compliance was assessed using the WHO’s “Your 5 Moments for Hand Hygiene” to define HH opportunities.^21^ HH compliance was calculated as the total number of observed HH practices (handwashing and alcohol-based hand rub use) divided by the total number of opportunities. For each professional category and care department, 200 opportunities were observed.

#### Healthcare waste management audit

An audit of healthcare waste management practices was conducted annually by the Department of Preventive and Community Medicine using a standardized assessment tool based on ANGED’s guidelines.^22^ All departments and units involved in healthcare delivery were included in the assessment, except those without direct care activities (eg, laundry room, sterilization unit, outpatient consultation areas, and internal and external pharmacies). The overall healthcare waste sorting indicator was calculated as the average of the following three criteria: absence of general waste in yellow bags, absence of infectious healthcare waste in black bags, and absence of infectious healthcare waste (other than sharps and cutting items) in sharps containers.

#### Personal protective equipement consumption

Data on alcohol-based hand rub and PPE consumption, including gloves, surgical masks, and FFP2 respirators, were obtained by the hospital’s internal pharmacy. Annual consumption of PPE and alcohol-based hand rub (liters/year) in 2021 was compared with pre-pandemic consumption in 2019.

### Statistical analysis

Categorical variables were expressed in frequencies and percentages, while continuous variables were expressed in means (standard deviations) or medians (Q1–Q3). To assess changes in HAI rates between the pre-pandemic and pandemic periods, we used the following formula for percentage change: (new value−old value)/old value∗100.

Comparisons across proportions were assessed using Fisher’s exact test. Comparisons between means were conducted using the independent samples *t* test in cases of normal distribution, and Mann–Whitney test was applied when the distribution was non-normal. Binary logistic regression analysis was performed to determinate risk factors associated with HAIs across the pre-COVID-19 and during COVID-19 pandemic periods. Variables with statistical significance (*p* < 0.2) in the univariate analysis were included in the final logistic regression models.^23^ All statistical analysis were performed using IBM SPSS Statistics 25.0 software (SPSS, Armonk, NY, USA). A 95% confidence interval (CI) and a *p*-value of 0.05 or less were considered statistically significant for all analyses.

## Results

Impact of the IPC Program on HAI Prevalence During the study period, a total of 534 patients were surveyed across 22 wards/units: 306 in the pre-COVID-19 period and 228 during the COVID-19 period. Most patients were male, accounting 59.2% of the pre-COVID-19 group and 53.5% of the COVID-19 group (*p* = 0.193). The median age was 51.0 years (IQR : 30.0–63.0) in 2019 and 53.5 years (IQR : 27.5–69.0) in 2021 (*p* = 0.482). Most patients were admitted to a medical department in 2019 (46.1%) and to a surgical department in 2021 (50.0%). Diabetes mellitus was the most common comorbidity in both periods (21.2% in 2019 vs 17.5% in 2021; *p* = 0.288). The proportion of patients receiving parenteral nutrition was significantly higher in 2019 (10.5%) than in 2021 (5.2%; *p* = 0.021). Antibiotic use in the previous 6 months increased from 15.4% (pre-COVID-19) to 27.2% (during COVID-19) (*p* = 0.001). There was no significant difference in the use of medical devices between the two periods (Table 1).

**Table 1.** Characteristics of patients admitted to Sahloul University Hospital of Sousse, Tunisia before and during the COVID-19 pandemic

Characteristics	Pre-COVID-19 (2019) Frequency (%) (*n* = 306)	During COVID-19 (2021) Frequency (%) (*n* = 228)	*p-V*alue*
**Age (year)**			
**Median (Q1-Q3)**	51.0 (30,0–63.0)	53.5 (27.5–69.0)	0.482
**Gender**			
*Male*	181 (59.2)	122 (53.5)	0.193
*Female*	125 (40.8)	106 (46.5)	0.193
**Ward of admission**			
*Surgical*	135 (44.1)	114 (50.0)	0.178
*Medical*	141 (46.1)	86 (37.7)	0.053
*ICU*	30 (9.8)	28 (12.3)	0.363
**Lenght of stay (days)**			
• *≤7*	144 (47.1)	122 (53.5)	0.14
• *8-30*	130 (42.5)	86 (37.7)	0.267
• *>30*	32 (10.5)	20 (8.8)	0.516
**Intrinsic risk factors**			
*Diabetes*	65 (21.2)	40 (17.5)	0.288
*Obesity*	10 (3.3)	23 (10.1)	**0.001**
*Immunodepression*	28 (9.2)	6 (2.6)	**0.002**
**Medical devices**			
*Urethral catheter*	62 (20.3)	57 (25.0)	0.193
*Mechanicl ventilation*	21 (6.9)	16 (7.0)	0.944
*Central venous catheter*	22 (7.2)	24 (10.5)	0.174
*Peripheral Catheter*	168 (54.9)	124 (54.4)	0.906
*Parenteral nutrition*	16 (5.2)	24 (10.5)	**0.021**
**Surgery within the past 30 days**	101 (33.0)	77 (33.8)	0.853
**Antibiotics-use in the last 6 months**	47 (15.4)	62 (27.2)	**0.001**
**Antibiotics intake on the day of the survey**	94 (30.7)	80 (35.1)	0.287

ICU: Intensive care unit, *Pearson’s χ^2^ test.

In the pre-COVID-19 period, HAI prevalence was 9.4% (29/306) compared to 10.1% (23/228) during the COVID-19 period (*p* = 0.814). By department, no statistically significant differences were observed.

In surgical wards, HAI prevalence increased from 6.7% (9/135) to 12.3% (14/114; *p* = 0.186), and in medical wards, it declined from 3.5% (5/141) to 1.2% (1/86; *p* = 0.412) (Table 2).

**Table 2.** Prevalence of hospital-acquired infections by department

Department	Pre-COVID-19 (n = 306)	During COVID-19 (n = 228)	*p-V*alue*
**Medical**	3.5% (5/141)	1.2% (1/86)	0.412
**Surgical**	6.7% (9/135)	12.3% (14/114)	0.186
**ICU**	40.0% (12/30)	25.0% (7/28)	0.270

ICU: Intensive care unit, *Pearson’s χ^2^ test.

Ventilator-associated pneumonia (VAP) was the most common infection pre-COVID-19 (2.6%), while surgical site infection (SSI) predominated during COVID-19 (2.6%). No significant differences were found for VAP (*p* = 0.296), urinary tract infections (UTIs; 1.3% vs 1.8%, *p* = 0.674), SSIs (1.6% vs 2.6%, *p* = 0.422), catheter-associated urinary tract infections (CAUTIs; 1.0% vs 1.3%, *p* = 0.716), and central-line-associated bloodstream infections (CLABSIs; 0.3% vs 1.8%, *p* = 0.090) between the two periods (Table 3).

**Table 3.** Frequency and type of hospital-acquired infections before and during the COVID-19 pandemic

Type of infection	Pre-COVID 19 (*n* = 306) Frequency (%)	During COVID-19 (*n* = 228) Frequency (%)	*p-V*alue*
VAP	8(2.6)	3(1.3)	0.296
UTI	4 (1.3)	4 (1.8)	0.674
SSI	5 (1.6)	6 (2.6)	0.422
CAUTI	3 (1.0)	3 (1.3)	0.716
CLABSI	1 (0.3)	4 (1.8)	0.090
BSI not CLABSI	3 (1.0)	2 (0.9)	0.903
Skin and soft tissue	1 (0,3)	0 (0)	0.388
Systemic infection	1 (0.3)	0 (0)	0.388
CNS	1 (0.3)	1 (0)	0.834S
Others	2 (0.6)	0 (0)	0.473

SSI, surgical site infection. VAP, ventilator-associated pneumonia. CLABSI, central line-associated bloodstream infections. CAUTIs, catheter-associated urinary tract infections. UTI, urinary tract infection. CNS, central nervous system. Others: oral cavity, *Pearson’s χ^2^ test.

Among patients with medical devices, 11.4% (12/105) developed device-associated infections pre-COVID-19, compared to10.3% (10/97) during the pandemic (*p* = 0.799). VAP remained the most prevalent device-associated infection in both periods (38.1% vs 18.8%, *P* = 0.202). Although CLABSI and CAUTI rates increased by 104% and 271%, respectively, these changes were not statistically significant (Table 4).

**Table 4.** Medical devices-associated infections before and during the COVID-19 pandemic

Medical devices site	Pre-COVID-19 (n = 105)	During COVID-19 (n = 97)	Percentage change (%)	*p*-Value*
VAP	8/21 (38.1%)	3/16 (18.8%)	−50.65	.202
CAUTIs	3/62 (4.8%)	3/57 (5.3%)	104.16	.916
CLABSIs	1/22 (4.5%)	4/24 (16.7%)	271.11	.187

VAP, ventilator-associated pneumonia. CLABSI, central line-associated bloodstream infections. CAUTIs, catheter-associated urinary tract infections. *Pearson’s χ^2^ test.

A total of 27 pathogens were isolated pre-pandemic, versus 24 during the pandemic. Pre-COVID-19, Pseudomonas aeruginosa (25.9%), Escherichia coli (18.5%), and Klebsiella pneumoniae (14.8%) predominated. During COVID-19, coagulase-negative staphylococci (20.8%) and Acinetobacter baumannii (16.6%) were most frequent. Coagulase-negative staphylococci increased significantly (0% vs 20.8%, *p* = 0.013), and E. coli decreased (18.5% vs 0%, *p* = 0.026). Enterobacter aerogenes and Providencia were detected only pre-pandemic; Enterococcus spp. and non-albicans Candida appeared only during the pandemic (Table 5).

**Table 5.** Type and frequency of microorganisms isolated in pre and during the COVID-19 pandemic

Family of microorganism		Microorganism	Pre-COVID 19 (%) (*n* = 27)	During COVID19 Frequency (%) (*n* = 24)		*p*-Value*
Gram Cocci positive		*Staphylococcus aureus* *Coagulase-negative staphylococci* *Streptococus pyogenes* *Others streptococcus (C, G, non groupables)* Enterococcus spp	2 (7.4) 0 1 (3.7) 1 (3.7) 0	2 (8.3) 5 (20.8) 1 (4.1) 0 1 (4.1)	0.902 0.013 0.932 0.341 0.284	
		*Corynebacteriaceae*	1 (3.7)	1 (4.1)	0.932	
Gram-negative bacillus	**Enterobacter**	*Klebsiella pneumoniae* *Enterobacter cloacae complex* *Proteus mirabilis* *Escherichia coli* *Enterobacter aerogenes* *Providencia*	4 (14.8) 1 (3.7) 0 5 (18.5) 1(3.7) 1 (3.7)	2 (8.3) 1 (4.1) 1 (4.1) 0 0 0	0.473 0.923 0.284 0.026 0.341 0.341	
	**Non** **enterobacter**	*Pseudomonas aeruginosa* *Acinetobacter baumanii*	7(25.9) 1 (3.7)	3 (12.5) 4 (16.6)	0.228 0.120	
Yeast		*Candida albicans*	2 (7.4)	1 (4.1)	0.632	
		*Candida non albicans*	0	2 (8.3)	0.126	

* Person’s χ2 test.

In multivariate analysis, diabetes mellitus was a significant HAI risk factor only pre-COVID-19 (OR = 5.24; 95% CI =1.34–20.48; *p* = 0.017), while recent surgery (within 30 days) was significant only during COVID-19 (OR = 11.97; 95% CI =2.54–56.23; *p* = 0.002). Antibiotic use in the last 6 months remained a risk factor in both periods (pre-COVID-19 OR = 9.50; 95% CI = 2.66–33.86; *p* = 0.001; COVID-19 OR = 3.43; 95% CI = 1.09–10.76; *p* = 0.035). Mechanical ventilation was independently associated with HAIs both before (OR = 17.01; 95% CI =3.80–76.06; p < 0.001) and during the pandemic (OR = 9.24; 95% CI = 2.41–35.48; *p* = 0.001). Central-line catheter use was a risk factor only pre-COVID-19 (OR = 17.58; 95% CI = 3.03–54.38; *p* = 0.001) (Table 6).

**Table 6.** Risk factors associated with hospital-acquired infections before and during the COVID-19 pandemic in multivariate analysis

Risk factors	Pre-COVID-19			During COVID-19		
	Adjusted OR	95% CI	*p*-Value	Adjusted OR	95%CI	*p*-Value
Diabetes mellitus	5.24	1.34–20.48	0.017	3.58	0.93–13.68	0.062
Antibiotics use in the last 6 months	9.50	2.66–33.86	0.001	3.43	2.66–33.86	0.035
Surgery within the past 30 days	0.80	0.22–2.84	0.733	11.97	2.54–56.23	0.002
Central vascular catheter	17.58	3.03–54.38	0.001	3.22	0.52–19.93	0.207
Mechanical ventilation	17.01	3.80–76.06	0.001	9.24	2.41–35.48	0.001

**OR**: odds ratio, statistically significant (*p* < 0.05).

Monitoring of IPC Compliance Global compliance with HH, as defined by the WHO “5 Moments,” increased from 38.3% in the pre-COVID-19 period to 51.6% during the COVID-19 period (*p* < 0.001). Compliance with alcohol-based hand rub use rose from 19.0% to 46.0% (*p* < 0.001). By professional category, physician compliance improved from 36.0% to 51.8% (*p* < 0.001), while nurse/technician compliance increased from 8.4% to 43.5% (*p* < 0.001). For healthcare waste management audit, the overall compliance rate for sorting sharps increased from 60.3% to 77.6%, while compliance for sorting soft and solid waste rose from 32.5 to 72.4% in 2021.

Regarding resource use, annual consumption of alcohol-based hand rub increased 3.22-fold, reaching 6,759 L in 2021. Similarly, the use of liquid disinfectants tripled, from 270 L to 800 L. PPE consumption (gloves, surgical masks, and FFP2 respirators) rose significantly, with FFP2 respirator use increasing nearly 247-fold—from 330 to 81,517 units annually (Table 7). there is a discrepnacy between the numbers and percentages in Table 7

**Table 7.** Monitoring of IPC measures before and during COVID-19 pandemic

Variables	Before COVID-19 pandemic (2019)	During COVID-19 pandemic (2021)
Hand hygiene		
Global hand hygiene compliance (%)	38.3	51.6
Global alcohol-based hand rub compliance (%)	19.0	46.0
*Alcohol-based hand rub* compliance by professionnels category (%)		
*Doctors*	36.0	51.8
*Nurses/technicians*	8.4	43.5
Alcohol-based hand rub consumption (Liters/year)	2,094	6,759
Personal protective equipment		
*Gloves (pairs/year)*	236,750	377,730
*Surgical mask (unit/year)*	123,200	839,520
*FFP2 respirators (unit/year)*	330	81,517
Environmental health		
Sorting and packaging steps audit (%)		
*Sharps (overall compliance rate)*	60.6	77.6
*Soft and solid waste (overall compliance rate)*	32.5	72.4
*Liquid isinfectant floors and surfaces (liters/year)*	270	800

## Discussion

HAIs represent a major public health concern worldwide, leading to increased morbidity, mortality, and healthcare costs.^24^ A lack of compliance with hospital safety and infection control guidelines has been identified as a leading cause of HAIs.^24^ This study found that the consumption of alcohol-based hand rub for HH and the use of PPE were significantly higher in 2021 compared to 2019. In addition, there was a marked increase in compliance with HH practices and healthcare waste management.

Despite these improvements, no statistically significant reduction in the prevalence of HAIs was observed—whether at the overall hospital level, by infection site, and by hospital department. These findings align with previous research suggesting that enhanced infection control strategies may not significantly affect overall HAI rates.^8,14,25–28^ A systematic review and meta-analysis conducted between 2019 and 2022 found that the COVID-19 pandemic did not alter the overall risk of HAI among hospitalized patients.^10^ Likewise, a multicenter study in the northeastern United States—analyzing over 20,000 total joint arthroplasties—reported no significant difference in early or late infection rates between pre-pandemic and pandemic cohorts, despite greater compliance with HH measures during COVID-19.^27^

Several factors may explain the lack of reduction in HAIs in our cohort. Before the pandemic, 46.1% of patients were admitted to medical wards, whereas during the pandemic, 50.0% were surgical wards. Undergoing surgery within the previous 30 days also emerged as a significant risk factor for HAIs during the COVID-19 period. A recent meta-analysis conducted in Africa confirmed that admission to surgical wards significantly increases HAI risk.^3^ Furthermore, a systematic review of COVID-19 countermeasures concluded that surgical patients benefit less from enhanced IPC strategies.^29^

In addition, the prevalence of patients receiving parenteral nutrition significantly increased during the COVID-19 period (*P* = 0.021), as did the proportion of patients with immunosuppression (*P* = 0.002). The reduction in scheduled procedures and outpatient consultations may have led to a delayed presentation of chronic patients (such as those with cancer or inflammatory diseases), often in a more malnourished condition. Similarly, the administration of high-dose corticosteroids in the management of severe cases may have contributed to mmunosuppressive states. Taken together, these factors may explain the absence of a significant decrease in HAI rates, despite improved compliance with respiratory precautions and HH practices.

Conversely, some reports have shown that improved IPC measures did reduce HAI rates during the pandemic.^13–32^ However, many of these studies were conducted in specialized units or smaller hospitals with fewer beds,^13,30^ or in “COVID-free” facilities that did not face the same surge in patient load.^29^ Others already had optimal IPC programs and low baseline HAI rates prior to COVID-19.^31,32^

Regarding device-associated infections, we observed a 50.6% reduction in VAP prevalence (*P* = 0.202), while CLABSI and CAUTI rates increased by 271% and 104%, respectively, although these increases were not statistically significant. These trends mirror reports of rising CLABSI ^8,33,34^ and CAUTI rates,^33^ with PPE and staffing shortages implicated as contributing factors.^33,34^ Among the studies, both staffing and PPE shortages have been identified as contributing factors to the increase in catheter-associated infections. To accommodate the growing number of patients and preserve limited PPE and staffing resources, the frequency of patient contact was often reduced during the pandemic. As a result, healthcare workers were encouraged to batch care tasks during each room visit to minimize PPE use. However, this approach may have increased the workload per visit and created more opportunities for lapses in HH compliance.^35^

By pathogen, Escherichia coli infections dropped to 0% during the COVID-19 period—a change attributed to enhanced IPC strategies, including HH—similar to observations by Gaspari et al.^36^ In contrast, SSI caused by coagulase-negative staphylococci increased significantly, likely driven by a higher volume and complexity of surgical cases during the pandemic.

Consistent with prior research,^7,8^ antibiotic use within the previous 6 months and mechanical ventilation remained significant risk factors for HAIs in both periods. Pre-COVID-19, diabetes mellitus and use of medical devices were significant factors; during COVID-19, recent surgery (within 30 days) became a predominant risk factor, underscoring the impact of invasive procedures on HAI prevalence.^7,8,36^

### Strengths and limitations

To the best of our knowledge, this is the first study discussing the effect of the IPC strategies implemented during the COVID-19 pandemic on HAI rates in low- and middle-income (LMIC) countries.^29^ A recent systematic review published in 2024 reported that no previous studies had addressed the potential beneficial effects of anti-COVID-19 measures on HAIs rates in LMIC.^29^ These findings may help improve the effectiveness of current IPC strategies and support healthcare systems in better preparing for future pandemics. However, this study has several limitations. First, although all eligible patients present in the hospital at the time of the survey were included, no significant difference in HAI rates was observed. This may be attributed to the small sample size, which may have limited the statistical power to detect such differences. Larger multicenter studies are needed to validate these findings and to support the continued application of preventive measures even after the pandemic to reduce HAI rates among hospitalized patients. Second, this study was based on annual PPS. Although incidence-based surveillance remains the gold standard for assessing the impact of IPC interventions on HAIs, in the absence of an active surveillance system, repeated standardized PPS can provide valuable insights into trends over time. Future research could benefit from incidence-based surveillance, particularly in high-risk units such as ICUs. Finally, the exclusion of COVID-19 patients may limit the generalizability of our findings and reduce comparability with other studies that included such patients.

## Conclusions

This study showed no significant changes in HAI prevalence despite the implementation of enhanced IPC strategies. However, the small sample size was the major limitation that may have reduced the statistical power to detect differences. Larger-scale studies are needed to confirm these findings and better assess the impact of IPC strategies in this context.

## Data Availability

The data sets generated and/or analysed during the current study are available from the corresponding author on reasonable request.
